# Case Report: Right pulmonary artery resection with artificial vascular graft replacement under cardiopulmonary bypass for pulmonary artery angiomatoid fibrous histiocytoma

**DOI:** 10.3389/fcvm.2026.1701193

**Published:** 2026-02-26

**Authors:** Tumin Sha, Jianqiang Li, Qian-li Wang, Chao Song, Xiaoxia Li, Chaoliang Liu

**Affiliations:** 1 The Second School of Clinical Medicine of Binzhou Medical University, Yantai Affiliated Hospital of Binzhou Medical University, Yantai, Shandong, China; 2 Department of Cardiac Surgery, Yantai Yuhuangding Hospital, College of Medicine, Qingdao University, Yantai, Shandong, China; 3 Department of Intensive Care Unit, Yantai Yuhuangding Hospital, College of Medicine, Qingdao University, Yantai, Shandong, China; 4 Department of Clinical Pathology, Yantai Yuhuangding Hospital, College of Medicine, Qingdao University, Yantai, Shandong, China

**Keywords:** angiomatoid fibrous histiocytoma, artificial vascular graft replacement, cardiopulmonary bypass, pulmonary artery tumor, transection of ascending aorta

## Abstract

This report describes an extremely rare case of primary right pulmonary artery angiomatoid fibrous histiocytoma (AFH). A 51-year-old female was admitted with “chest tightness and dizziness for over two months, aggravated by chest pain for three days”. Contrast-enhanced chest CT revealed an irregular filling defect (approximately 2.9 × 1.8 cm) in the right trunk of the pulmonary artery. The tumor in the right pulmonary artery was completely resected, followed by artificial vascular graft replacement under cardiopulmonary bypass with transection of the ascending aorta. Pathological examination confirmed AFH, with fluorescence *in situ* hybridization showing MDM2 (−, no amplification) and EWSR1 (+, break). No recurrence or metastasis of the tumor was observed during the five-month follow-up. Transection of the ascending aorta under cardiopulmonary bypass provided optimal exposure, enabling complete resection of the tumor while preserving right lung function through artificial vascular graft reconstruction, thereby achieving satisfactory clinical outcomes. This approach offers an effective surgical strategy for the management of pulmonary artery tumors, including AFH.

## 1 Introduction

Angiomatoid fibrous histiocytoma (AFH) is a rare neoplasm with an incidence of only 0.001%. It occurs predominantly in superficial soft tissues of the extremities and retroperitoneum in children and adolescents, and is exceedingly uncommon in extraskeletal soft tissues (1). There are only six cases of primary pulmonary artery AFH documented in the literature, with surgical approaches primarily including pneumonectomy, lobectomy, or tumor resection combined with pulmonary artery reconstruction. This report describes a case in which transection of the ascending aorta under cardiopulmonary bypass (CPB) support was employed to achieve optimal exposure, enabling complete resection of the tumor in the right pulmonary artery together with the involved segment of the pulmonary artery, followed by reconstruction using a synthetic vascular graft. There are few reports of this surgical technique in the literature.

## 2 Case presentation

**Figure 1:**
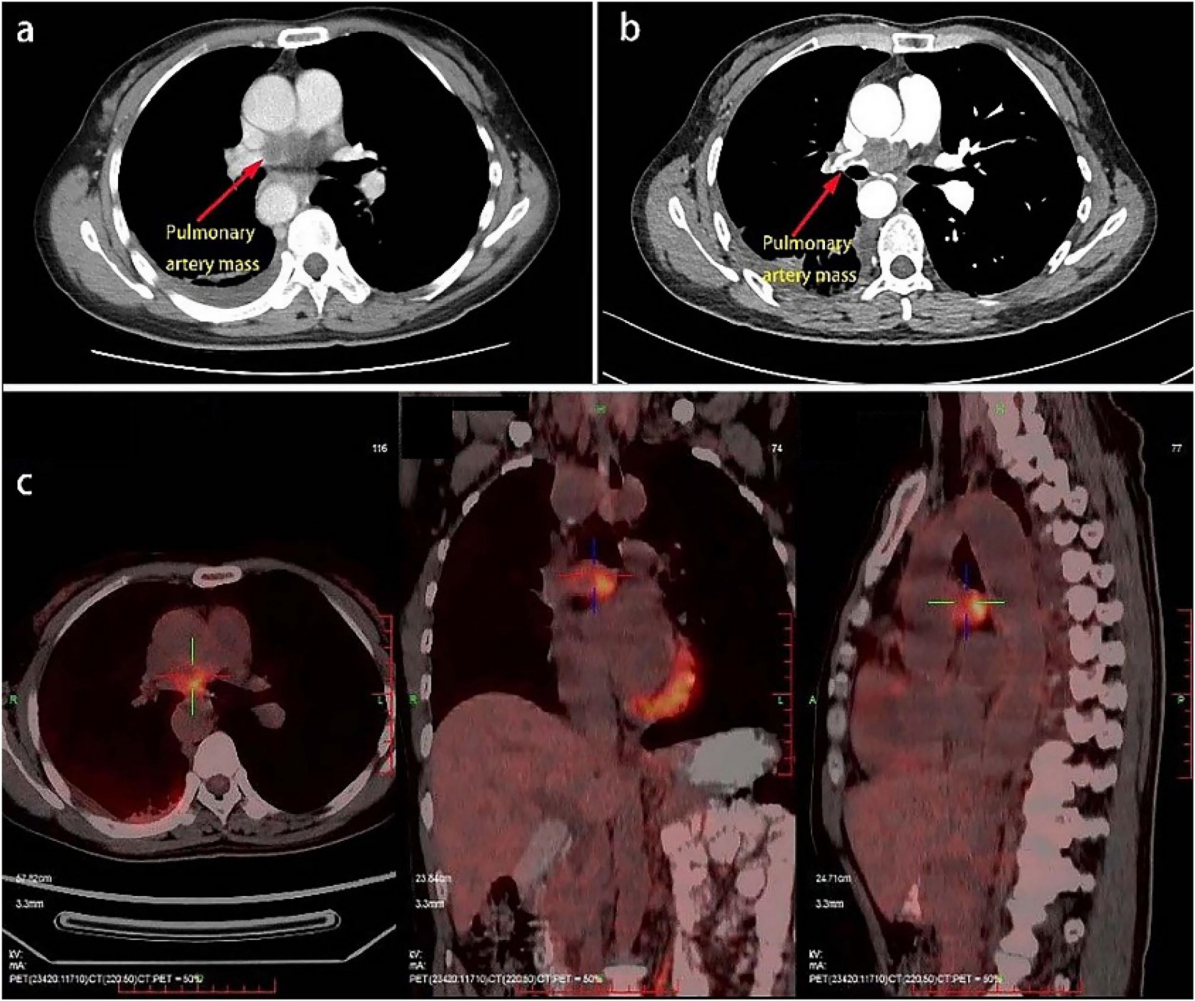
**(a)** chest CT shows an irregular filling defect in the right pulmonary artery trunk; **(b)** CTA of the thoracoabdominal aorta reveals a filling defect in the right pulmonary artery trunk, with involvement of the right upper lobe artery and right interlobar artery, distal narrowing, and sparse branching; **(c)** PET-CT demonstrates a patchy low-density shadow in the right pulmonary artery, measuring approximately 2.3*1.8*1.4 cm, with increased FDG uptake (SUVmax ∼10.0). No significant enlargement is observed in the mediastinal or bilateral hilar lymph nodes.

A 51-year-old female presented with “chest tightness and dizziness for over two months, aggravated by chest pain for three days”. Laboratory tests revealed hemoglobin 104 g/L, leukocytes 7.35 × 10^9^ /L, platelets 302 × 10^9^ /L, D-dimer 2.0 mg/L, fibrinogen 7.76 g/L, with normal levels of tumor markers. Contrast-enhanced chest CT demonstrated an irregular filling defect in the trunk of the right pulmonary artery with mild enhancement (Figure 1a). Pulmonary CTA showed irregular filling defects involving the right upper lobe and interlobar arteries (Figure 1b). PET-CT indicated the presence of a hypodense lesion (2.3 × 1.8 × 1.4 cm) with increased FDG uptake (SUVmax of approximately 10.0) (Figure 1c).

**Figure 2:**
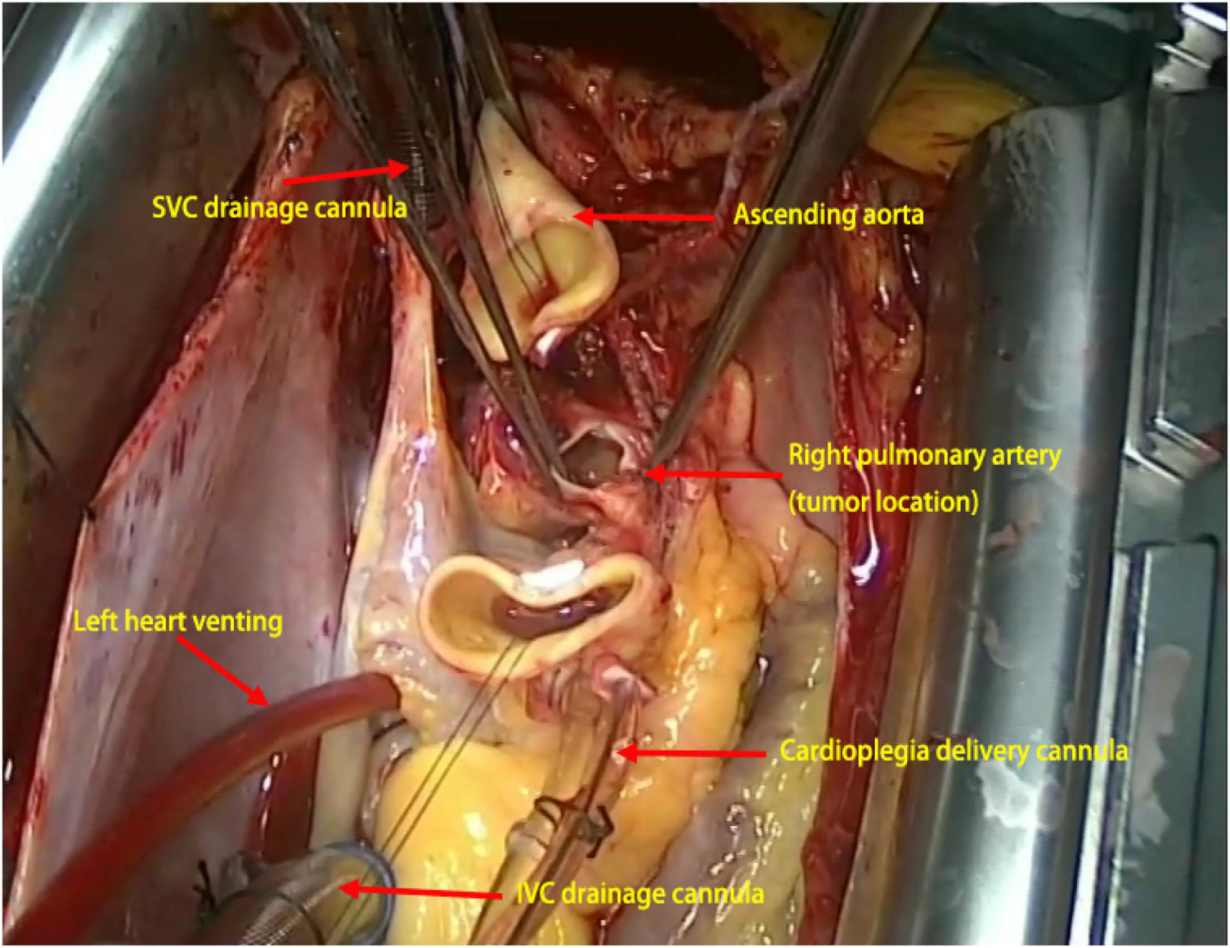
The intraoperative exposure: the ascending aorta was transected and retracted, providing optimal exposure of the right pulmonary artery tumor, the artery itself, and the surrounding tissues. Left heart venting (cannulation site: right superior pulmonary vein); SVC, superior vena cava; IVC, inferior vena cava.

**Figure 3:**
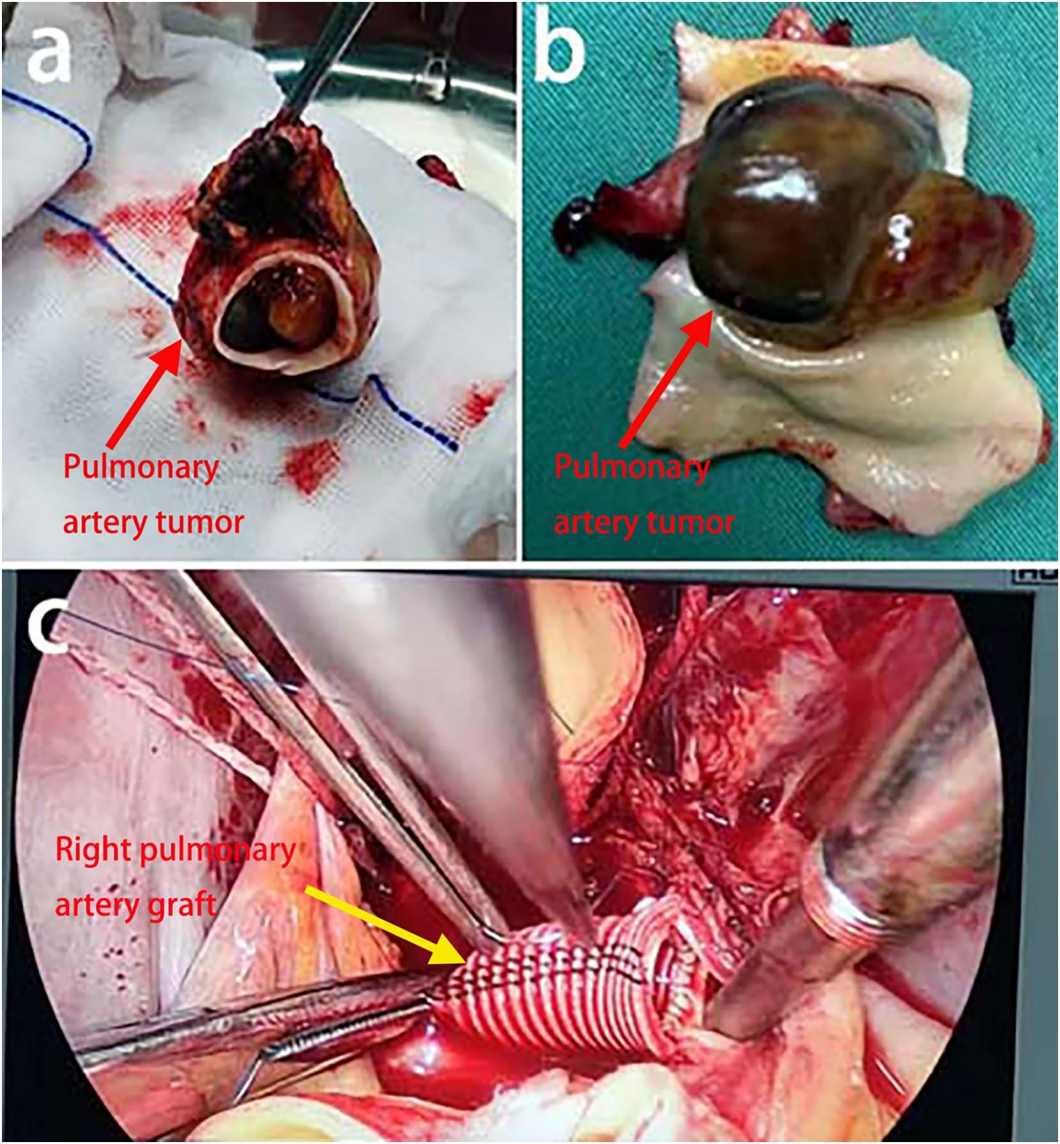
**(a)** the tumor was completely excised. **(b)** The tumor measured 4.5 × 3 × 2.5 cm, with a smooth, grayish-red to dark-brown surface and soft consistency, and was attached to the inferior wall with a 3 × 2.5 × 1.5 cm pedicle; **(c)** An artificial vascular graft was anastomosed to both the root of the right pulmonary artery and its first branch.

Right femoral arterial cannulation and superior/inferior vena caval cannulation were performed to institute cardiopulmonary bypass. After aortic cross-clamping and caval snaring, cardioplegia was delivered, and a catheter was advanced across the patent foramen ovale to decompress the left heart. The ascending aorta was transected 1 cm distal to the clamp and retracted, providing an unobstructed view of the right pulmonary artery tumour (Figure 2). The intraoperative findings included a thinned wall of the right pulmonary artery with extensive adhesions. After complete mobilization, the tumor and the right pulmonary artery were resected *en bloc* from the origin of the artery to 5 mm proximal to the first branch (Figures 3a,b). The proximal and distal arterial margins were immediately submitted for intraoperative frozen-section evaluation; the pathologist reported no tumor cells at either margin. No abnormalities were observed in the remaining pulmonary artery. An INTERGARD WOVEN 10 mm × 5 cm artificial graft was implanted (Figure 3c), followed by reconstruction of the ascending aorta and successful weaning from CPB.

**Figure 4:**
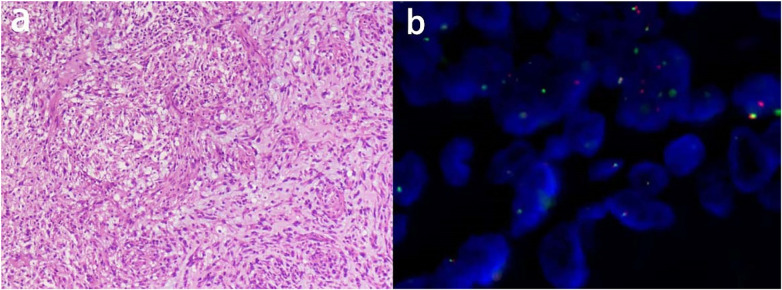
**(a)** H&E, 10 × 10. (Pulmonary artery mass) Tumor cells are spindle-shaped, with mild-moderate atypia, stromal myxoid degeneration and infiltration of lymphocytes and plasma cells around the periphery; **(b)** Fluorescence *in situ* hybridization, ×1,000. MDM2 (−, no amplification); EWSR1 (+, breakage).

**Figure 5:**
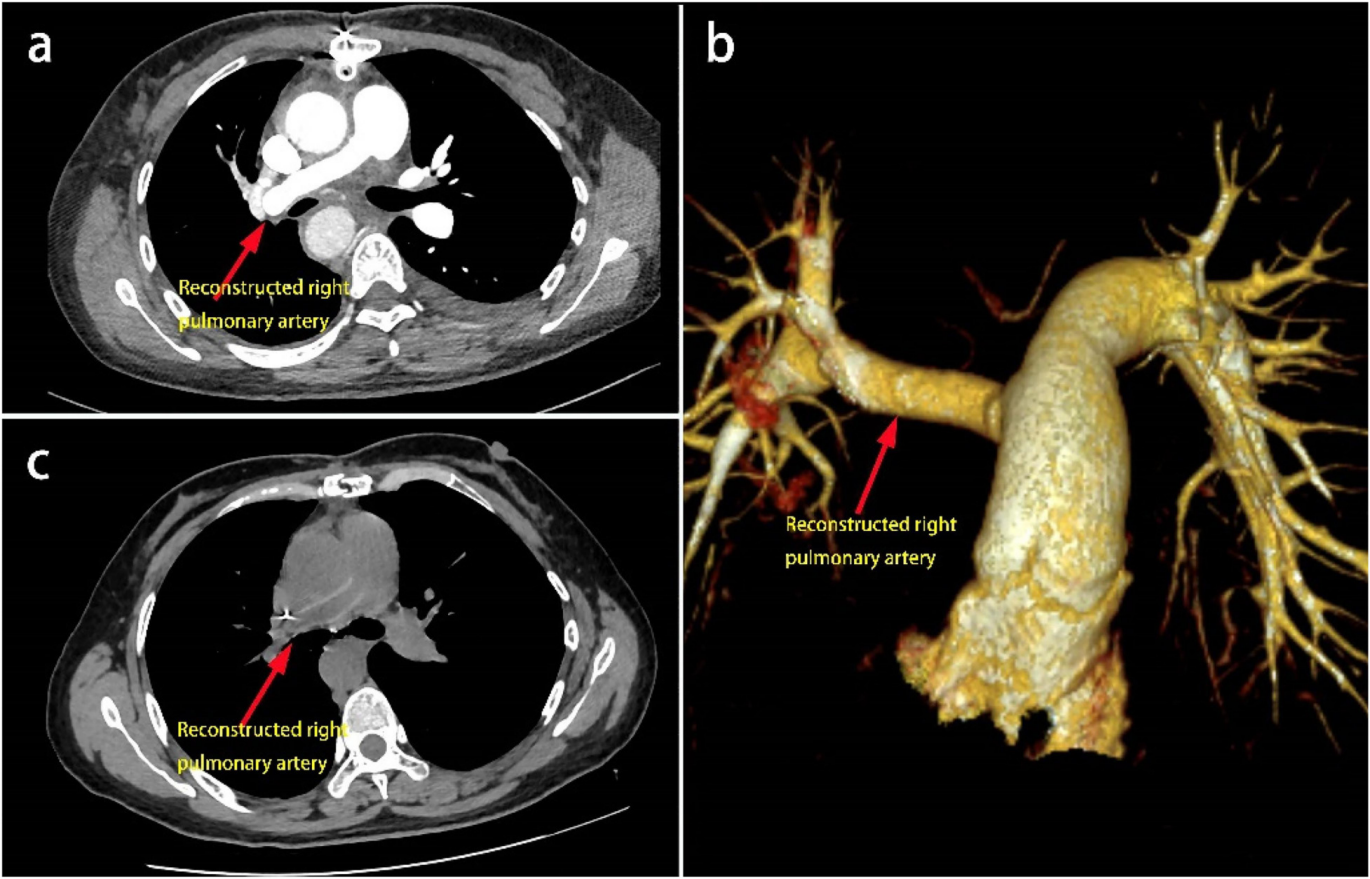
**(a,b)** follow-up chest CTA demonstrates patency of the artificial vascular graft with no significant filling defect observed; **(c)** Two-month postoperative chest CT reveals that the right pulmonary artery trunk is narrower than the contralateral side, but no obvious filling defect is seen within it.

Pathological examination of the tumor revealed the presence of spindle-shaped tumor cells with mild-to-moderate atypia, myxoid stroma, and peripheral lymphoplasmacytic infiltration (Figure 4a), consistent with AFH. Immunohistochemistry showed Vimentin (+), Desmin (focal weak +), EMA (focal +), CD99 (scattered +), and Ki-67 (+, 5%). FISH demonstrated MDM2 (−) and EWSR1 (+) (Figure 4b). CTA on postoperative day 6 confirmed a patent graft (Figures 5a,b). Follow-up CT at 5 months showed no recurrence or metastasis (Figure 5c).

## 3 Discussion

Since Enzinger (2) first proposed the concept of AFH in 1979, further research has led to its current classification as an intermediate malignancy. However, its postoperative recurrence rate is approximately 15%, with metastasis and mortality rates around 1% (3). Notably, “visceral-type” AFH (occurring in the brain, mediastinum and lungs, for example) tends to be associated with larger tumor sizes and a higher likelihood of postoperative recurrence (4, 5).

The preoperative diagnosis of pulmonary artery AFH is extremely challenging due to the lack of specific clinical manifestations. A review of reported cases indicated that approximately 71% (5/7) of patients were initially misdiagnosed with pulmonary embolism, as was the present case (5). Preoperative biopsy, although diagnostically possible, is generally discouraged due to challenges in sampling and high risks. The use of PET/CT has reduced the misdiagnosis of pulmonary artery tumors, as uptake of 18F-FDG by the tumor provides an effective diagnostic clue (6).

There is no unified treatment guideline for pulmonary artery AFH (4). Table 1 summarizes the six previously reported cases of pulmonary artery AFH, along with our own case. Depending on the location of AFH within the pulmonary artery, the surgical strategies used have included pneumonectomy, lobectomy, and tumor resection with pulmonary artery reconstruction (4, 5, 7–10). Pneumonectomy or lobectomy can impair pulmonary function, impacting both short- and long-term quality of life. Tumor resection with pulmonary artery reconstruction is only feasible for small tumors with minimal invasion of the surrounding tissue. In the present case, the tumor was located in the trunk of the right pulmonary artery, extending into the anterior segment of the first branch of the artery. The deep location, vascular wall invasion of the tumor, as well as the presence of severe adhesions to surrounding tissues, would have made tumor exposure using conventional thoracic surgical approaches extremely difficult. This would preclude both complete resection of the tumor and lymph node dissection.

**Table 1:** Retrospective analysis of surgical data in patients with pulmonary artery AFH.

Case	Sex	Age(years)	Primary site	Type of surgery	Follow-up
Ghigna et al. (4)	Female	76	RPA	Resection of 3 cm of the RPA + end-to-end anastomosis	/
Munir et al. (5)	Male	20	LPA	Resection of the LPA + vascular reconstruction	Disease-free survival at 12 months post-surgery
Mishima et al. (7)	Male	42	RSPA	Right upper lobectomy	Disease-free survival at 6 months post-surgery
Farag et al. (8)	Female	62	LPA	Left pneumonectomy	No postoperative complications at 7 days
Haug et al. (9)	Male	39	RIA	Right pneumonectomy	/
Chen et al	Male	52	LPA、LSPA	Left pneumonectomy	Disease-free survival at 7 months post-surgery
Present case	Female	51	RPA	Resection of 5 cm of the RPA + prosthetic graft replacement	Disease-free survival at 5 months post-surgery

**/**, indicates missing information; RPA, right pulmonary artery trunk; RSPA, right superior pulmonary artery; LPA, left pulmonary artery trunk; RIA, right interlobar artery; LSPA, left superior pulmonary artery.

We therefore employed a refined strategy that couples cardiopulmonary bypass with transection of the ascending aorta, thereby overcoming the exposure constraints of standard thoracic approaches. This allowed optimal exposure of the tumor in the right pulmonary artery, as well as the artery itself and the surrounding tissues, enabling complete tumor resection, thorough lymph node dissection, and the removal of potentially infiltrated tissues, thereby achieving precise radical tumor resection. Resection of the tumor-invaded pulmonary artery segment followed by its reconstruction using a synthetic graft circumvented the need for lobectomy or pneumonectomy, which would have been unavoidable in cases of large tumors or extensive vascular invasion. The transaortic technique was originally popularised for resecting primary sarcomas of the pulmonary trunk and mid-mediastinal tumours that cannot be safely exposed through a standard transverse sinus window (11–13). By dividing the ascending aorta under total CPB, the posterior aspect of the pulmonary bifurcation—especially the right pulmonary artery—is brought into a direct anterior field, allowing complete circumferential dissection without retractor-related haemodynamic compromise. We therefore adopted this approach because the tumour originated from the right pulmonary artery trunk and was densely adherent to the posterior aortic wall, rendering conventional lateral thoracotomy or median sternotomy insufficient without aortic transection for R0 resection.

Intraoperative frozen section confirmed tumour-free pulmonary artery margins, allowing us to proceed with prosthetic reconstruction while sparing the entire right lung and achieving an R0 resection. Paraffin validation remained negative, corresponding to disease-free survival at five months. For rare, deeply seated vascular tumours, seamless multidisciplinary teamwork—especially real-time dialogue with pathology—and rigorous intraoperative margin assessment are essential to avoid unnecessary pneumonectomy.

## 4 Conclusion

In summary, the adoption of CPB-supported transection of the ascending aorta circumvented the limitations of conventional thoracic surgery and enabled successful management of this exceptionally rare pulmonary-artery AFH. It offers a proven alternative strategy for patients with pulmonary artery tumors, including AFH.

## Data Availability

The original contributions presented in the study are included in the article/Supplementary Material, further inquiries can be directed to the corresponding authors.
